# The Pox in the North American Backyard: Volepox Virus Pathogenesis in California Mice (*Peromyscus californicus*)

**DOI:** 10.1371/journal.pone.0043881

**Published:** 2012-08-28

**Authors:** Nadia F. Gallardo-Romero, Clifton P. Drew, Sonja L. Weiss, Maureen G. Metcalfe, Yoshinori J. Nakazawa, Scott K. Smith, Ginny L. Emerson, Christina L. Hutson, Johanna S. Salzer, Jeanine H. Bartlett, Victoria A. Olson, Cody J. Clemmons, Whitni B. Davidson, Sherif R. Zaki, Kevin L. Karem, Inger K. Damon, Darin S. Carroll

**Affiliations:** 1 Poxvirus and Rabies Branch, Division of High-Consequence Pathogens and Pathology, National Center for Emerging and Zoonotic Infectious Diseases, Centers for Disease Control and Prevention, Atlanta, Georgia, United States of America; 2 Infectious Disease Pathology Branch, Division of High-Consequence Pathogens and Pathology, National Center for Emerging and Zoonotic Infectious Diseases, Centers for Disease Control and Prevention, Atlanta, Georgia, United States of America; 3 Program in Population Biology, Ecology, and Evolution, Emory University, Atlanta, Georgia, United States of America; Metabiota, United States of America

## Abstract

*Volepox virus* (VPXV) was first isolated in 1985 from a hind foot scab of an otherwise healthy California vole (*Microtus californicus*). Subsequent surveys in San Mateo County, CA, revealed serological evidence suggesting that VPXV is endemic to this area, and a second viral isolate from a Pinyon mouse (*Peromyscus truei*) was collected in 1988. Since then, few studies have been conducted regarding the ecology, pathology, and pathogenicity of VPXV, and its prevalence and role as a potential zoonotic agent remain unknown. To increase our understanding of VPXV disease progression, we challenged 24 California mice (*Peromyscus californicus*) intranasally with 1.6×10^3^ PFU of purified VPXV. By day five post infection (pi) we observed decreased activity level, conjunctivitis, ruffled hair, skin lesions, facial edema, and crusty noses. A mortality rate of 54% was noted by day eight pi. In addition, internal organ necrosis and hemorrhages were observed during necropsy of deceased or euthanized animals. Viral loads in tissues (brain, gonad, kidney, liver, lung, spleen, submandibular lymph node, and adrenal gland), bodily secretions (saliva, and tears), and excretions (urine, and/or feces) were evaluated and compared using real time-PCR and tissue culture. Viral loads measured as high as 2×10^9^ PFU/mL in some organs. Our results suggest that VPXV can cause extreme morbidity and mortality within rodent populations sympatric with the known VPXV reservoirs.

## Introduction

The genus *Orthopoxvirus* (OPXV) is the most important member of the family *Poxviridae* in terms of public health and includes viruses associated with severe febrile, rash illness in humans: *Variola virus, Monkeypox virus, Vaccinia virus, and Cowpox virus* [1], [2], [3], [4], [5]. The last few decades have seen the description of three OPXVs from North America named after the mammal species in which they were originally isolated: *Raccoonpox virus, Skunkpox virus,* and *Volepox virus* [6], [7], [8], [9], [10], [11]. Subsequent work has determined that the North American OPXV (NA OPXV) species are a monophyletic group which is the most genetically divergent within the OPXV genus [8].

*Volepox virus* (VPXV) was first isolated in June of 1985 from a hind foot scab of a healthy California vole (*Microtus californicus*) in San Mateo County, CA [10]. Serological evidence for the endemicity of VPXV in the San Francisco Bay region was obtained through testing (hemagglutinin inhibition antibody titers) vole serum sampled between 1983 and 1986 from separate populations in Marin, Santa Clara, and San Mateo counties [10]. A second identical isolate was obtained from a Pinyon mouse (*Peromyscus truei*) scab in 1988 on the Jasper Ridge Biological Preserve suggesting that the virus is endemic to this region [9]. Few studies have been conducted regarding the ecology, pathology and pathogenicity of VPXV; and its prevalence and role as a potential zoonotic agent remains unknown. Our study investigates the pathogenicity of VPXV within the California mouse (*P. californicus*), a rodent species that often occurs sympatrically with the presumed rodent reservoirs of VPXV.

## Results

### Morbidity and Mortality

**Figure 1:**
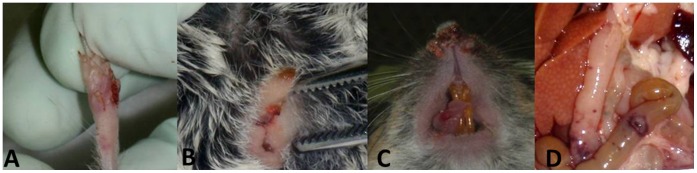
Characteristic lesions observed on *P.californicus* infected with Volepox virus. Epidermal ulcers with crusting on paw (A), vulvae and perineum (B), tongue and nares (C). Serosal petechiae and focal gastrointestinal tract necrosis (D).

By day five, several clinical signs (e.g., conjunctivitis, decreased activity level, ruffled hair, crusty noses, and facial edema) were observed in most of the animals along with the onset of “pox-like” skin lesions. Several 1–3 mm diameter epidermal hyperemias on tails were observed; maculae and papulae were present on eyelids, paws, ears, lips, and oral commissures. On day six, three mice had succumbed to disease, and three more were euthanized based on the clinical scale criteria outlined in the methodology section. On day seven, two additional mice had expired and three more were euthanized. Skin maculae and papulae progressed to erosions, and petechiae were observed on internal organs during necropsy of the deceased and euthanized animals (Fig. 1). On day eight, two more animals expired which generated a final mortality rate of 54% (13/24). The eleven (7/11 males and 4/13 females) VPXV-challenged, surviving mice had no observable signs of disease by day 12. Sex was not significantly related to survivorship (p = 0.07). The seven negative control animals gained weight during the study and statistical comparisons of mean weight loss between infected mice and negative controls were highly significant (p = 0.006). Changes in body temperatures were not as marked as the observed weight loss, but still significant (p = 0.03). The most substantial weight loss was observed on days five through seven which coincided with a decrease in body temperatures. On day twenty one, one survivor (*Peromyscus californicus* (PC) 015) was euthanized to determine if the animal had cleared infection. The rest of the California mice (10 animals) recovered uneventfully from VPXV infection and were included in an oral rabies recombinant vaccine study; they were euthanized at days 35 (n = 2), 42 (n = 3), 49 (n = 3) or 56 (n = 2) post VPXV infection.

### Viral DNA and Infectious Virus Observations

**Figure 2:**
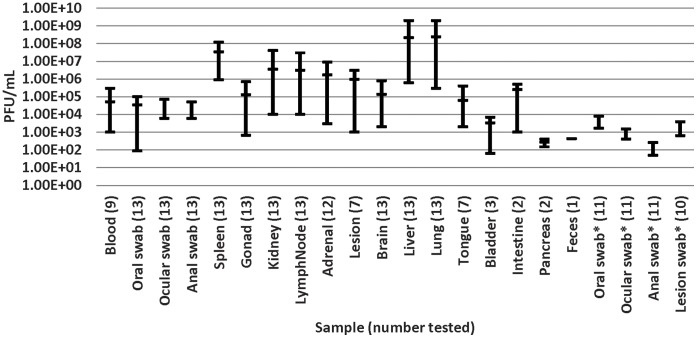
Viable virus content per specimen. Volepox virus loads (PFU/mL) per specimen taken during the necropsy of deceased and euthanized mice on days 6, 7 and 8 pi. *Swabs from the survivors were taken on day 7 pi.

Multiple tissues, secretions, and excretions collected on days six, seven, and eight generated CT values (the cycle when fluorescence crosses the threshold) between 15 to 40 for the NA OPXV real time-polymerase chain reaction assay (RT-PCR), indicating the presence of DNA specific for VPXV. The animal that was euthanized on day 21 had VPXV DNA (CT 40) only in brain tissue, and no viable virus was recovered. Oral, ocular, and anal swabs of the 11 survivors were RT-PCR negative by day 21. Samples collected throughout the study which had a CT value of 37 or below, equating to a minimum of 45 genomes of VPXV DNA (based on the standard curve), demonstrated detectable CPE in a single passage on BSC-40 cells. Viable virus could not be obtained after 48 hrs from specimens with a CT of ≥38. The highest observed viral titers from oral swabs were similar on days 6, 7, and 8 (1×10^5^, 3×10^4^, and 7×10^4^ PFU/mL respectively) as were viral titers in the spleen and liver samples on days 6 and 7 (1.2×10^8^, 4×10^8^, 1×10^8^, and 2×10^9^ PFU/mL respectively). The viral titer in the spleen and liver samples on day 8 had a decrease of 2–3 logs compared with day 6 and 7. On day 6, the lung viral titer measured up to 1.9×10^9^, but decreased two logs on days 7 and 8 (Fig. 2).

### Humoral Immune Response

Sixteen out of twenty-four (16/24) infected California mice sera were positive by enzyme-linked immunosorbent assay (ELISA), and 20/24 mice sera were positive by western blots (WB). Four animals (PC 010, 017, 024, and 027) did not have evidence of antibody production by either ELISA or WB. The remaining animals showed evidence of immune response in at least one assay (Table 1). The molecular weight protein bands observed in WB from animals which succumbed during the early phase of infection (6 to 8 days) were 18, 21, 36, 39, and 62 kilo-Daltons (kDa). In animals euthanized on or after day 21 (21 to 56 days), bands weighting 11, 14, 18, 21, 25, 32, 36, 62, and 75 kDa were observed. The 36 kDa band was immunodominant (present in >50%) and appeared in 7/13 mice that succumbed to the infection. The 14 kDa band was present in 9/9 survivors that manifest humoral immune response; two of eleven survivors were negative in both the ELISA and WB assays. The 36 and 62 kDa bands were also prominent in survivors; these bands were present in 6/9 and 8/9 which manifest an immune response, respectively. Furthermore, bands 11, 14, 25, 32, and 75 kDa were observed only in mice that survived on or past day 21 after VPXV infection (Table 2).

**Table 1:** Correlation between volepox virus infection and immune response.

Animal ID	Necropsy *	Cause of death	Inoculum	Lesions or signs of disease	ELISA **	Western Blot ***
PC 007	Day 6	D	VPXV 1.6 e3	paw, tongue, vulvae, liver	POS	18
PC 011	Day 6	D	VPXV 1.6 e3	conjuntivitis, vulvae, tongue	NEG	36,18
PC 012	Day 6	E	VPXV 1.6 e3	intestine, ovaries	POS	36,18
PC 014	Day 6	D	VPXV 1.6 e3	tail, tongue	POS	62,36,21
PC 020	Day 6	E	VPXV 1.6 e3	tongue, lip, nose	NEG	62,36,21
PC 030	Day 6	E	VPXV 1.6 e3	tail, tongue	POS	18
PC 008	Day 7	E	VPXV 1.6 e3	liver	NEG	36
PC 018	Day 7	E	VPXV 1.6 e3	tongue, intestine	NEG	62
PC 024	Day 7	D	VPXV 1.6 e3	paw, eyelid, intestine, liver	NEG	NEG
PC 027	Day 7	D	VPXV 1.6 e3	ear, tongue, intestine, kidney	NEG	NEG
PC 034	Day 7	E	VPXV 1.6 e3	submandibular lymph node	POS	62,36,21
PC 022	Day 8	D	VPXV 1.6 e3	lip, adrenal gland	POS	39,36,18
PC 037	Day 8	D	VPXV 1.6 e3	none	POS	62,21,18
PC 015	Day 21	S	VPXV 1.6 e3	skin	POS	62,36,25,21,14,11
PC 009	Day 35	S	VPXV 1.6 e3	skin	POS	62,36,14
PC 013	Day 35	S	VPXV 1.6 e3	tail	POS	62,36,14
PC 010	Day 42	S	VPXV 1.6 e3	skin	NEG	NEG
PC 016	Day 42	S	VPXV 1.6 e3	eyelid	POS	62,32,14
PC 021	Day 42	S	VPXV 1.6 e3	skin	POS	62,25,21,18,14,11
PC 017	Day 49	S	VPXV 1.6 e3	tail	NEG	NEG
PC 023	Day 49	S	VPXV 1.6 e3	tail	POS	75,62,36,32,25,21,18,14
PC 029	Day 49	S	VPXV 1.6 e3	tail	POS	62,36,14,11
PC 026	Day 56	S	VPXV 1.6 e3	crusty nose	POS	25,21,18,14
PC 032	Day 56	S	VPXV 1.6 e3	none	POS	62,36,14
PC 038	Day 7	S	PBS	none	NEG	NEG
PC 039	Day 14	S	PBS	none	NEG	NEG
PC 040	Day 21	S	PBS	none	NEG	NEG
PC 041	Day 28	S	PBS	none	NEG	NEG
PC 061	Day 35	S	PBS	none	NEG	NEG
PC 074	Day 42	S	PBS	none	NEG	NEG
PC 081	Day 49	S	PBS	none	NEG	NEG

* Days post infection.

** POS, positive.NEG, negative.

*** Protein band observed, size in kDa. NEG, no band observed.

D, die due to infection. E, met the euthanasia criteria. S, study scheduled euthanasia.

**Table 2:** Presence of western blot bands (kDa) in California mice infected with volepox virus.

Non survivors											
*Day	° ID #	11	14	18	21	25	32	36	39	62	75
6	PC 007			P							
6	PC 011			P				P			
6	PC 012			P				P			
6	PC 014				P			P		P	
6	PC 020				P			P		P	
6	PC 030			P							
7	PC 008							P			
7	PC 018									P	
7	∼PC 024										
7	∼PC 027										
7	PC 034				P			P		P	
8	PC 022			P				P	P		
8	PC 037			P	P					P	
Survivors											
21	PC 015	P	P		P	P		P		P	
35	PC 009		P					P		P	
35	PC 013		P					P		P	
42	∼PC 010										
42	PC 016		P				P			P	
42	PC 021	P	P	P	P	P				P	
49	∼PC 017										
49	PC 023		P	P	P	P	P	P		P	P
49	PC 029	P	P					P		P	
56	PC 026		P	P	P	P					
56	PC 032		P					P		P	

* Day post infection.

° Mouse identificacion number. P, present.

∼ Mice did not show evidence of immune response.

### Hematology

Animals that died between days six and eight had monocytosis with values measuring between: 0.89–1.3/L (normal range of 0–0.33×10^9^/L). These animals were also neutrophilic with values measuring between: 4.3–6.04/L (normal range of 0.07–2.7×10^9^/L). PC 015 showed an increase in the mean corpuscular volume (MCV) on day 21(75 fL when the normal range is 45–55 fL). Additionally, four mice that survived infection were sampled on day 28 and corroborated an increase of the MCV of 72–74 fL. Animals sampled from day six to 21 showed thrombocytopenia with values measuring between: 115–181/L (normal range of 200–450 ×10^9^/L).

### Pathological Findings

**Figure 3:**
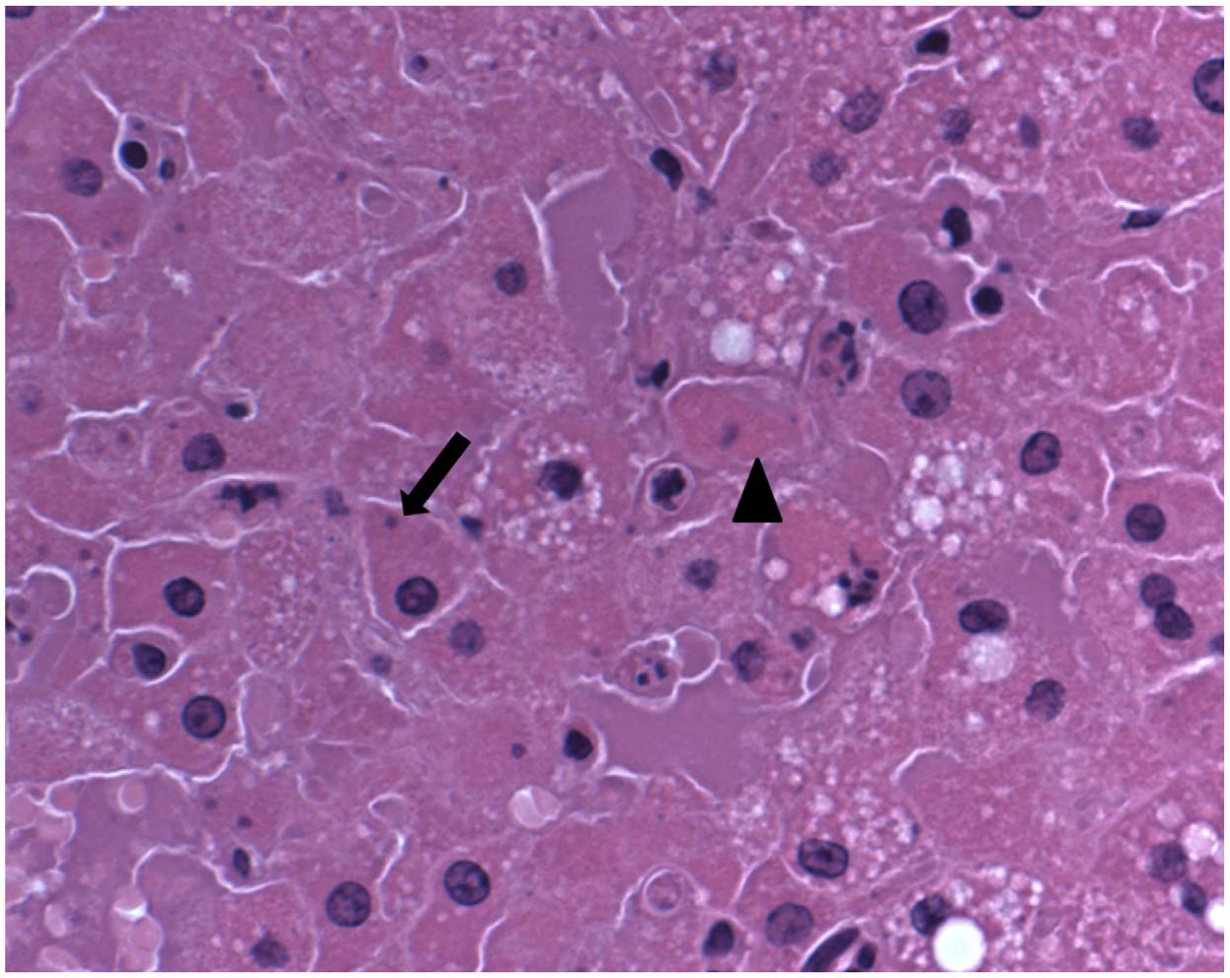
Hematoxylin-eosin stain of liver tissue. The arrow head indicates an example of cell necrosis (hepatocytes with homogeneously eosinophilic, or pink, cytoplasm and pyknotic or karyolytic nuclei). Hepatocytes occasionally have small basophilic intracytoplasmic inclusions observed in dark blue (arrow).

**Figure 4:**
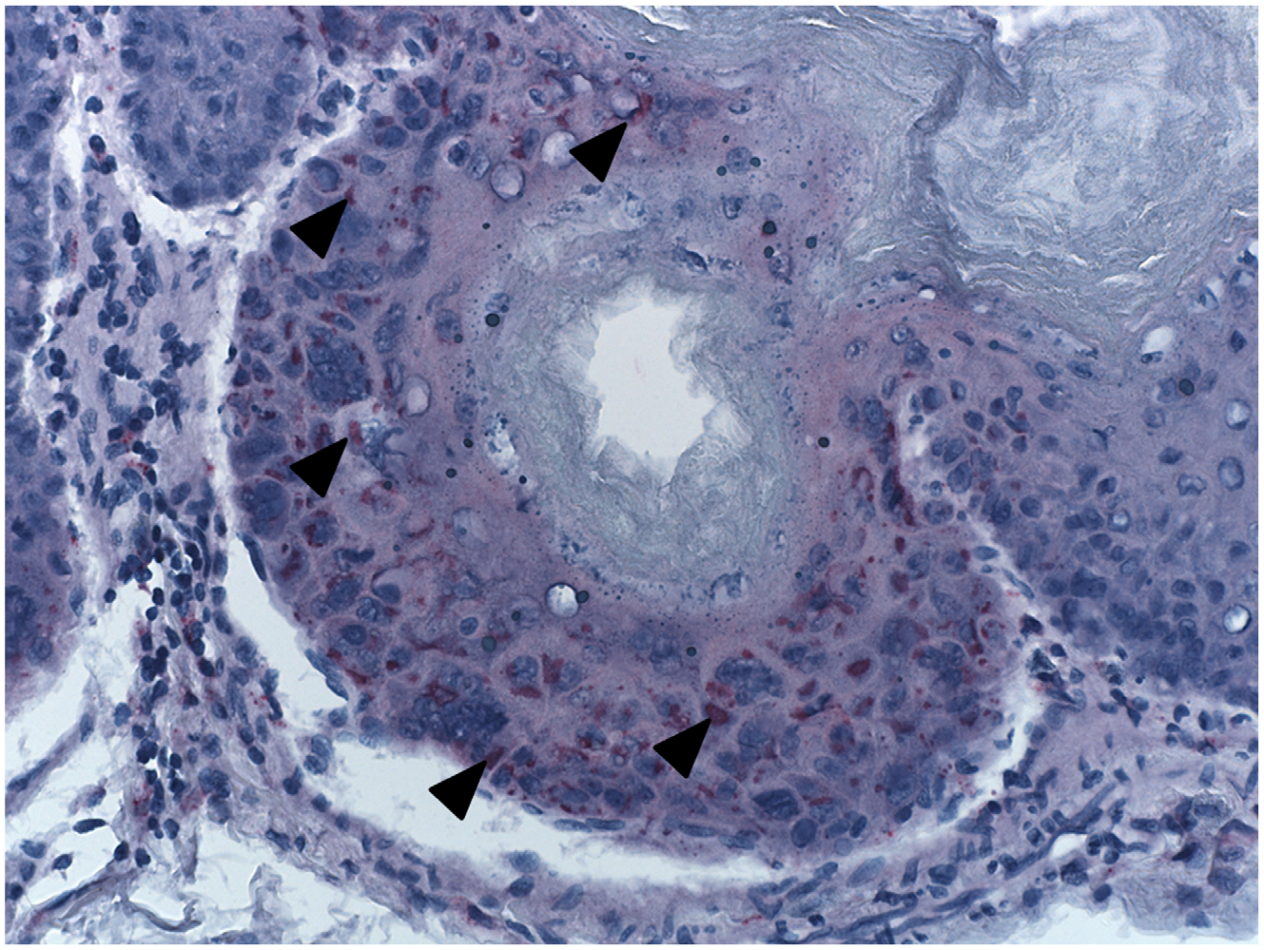
Immunohistochemical test results showing abundant volepox antigen. The antigen stained in red (arrow heads) within epithelial cells of a stomach specimen.

California mice exhibited histologic changes attributable to active virus infection (day 6 to 8) in the liver, gastrointestinal tract, adrenal gland, spleen, and lungs. The liver demonstrated single cell necrosis and abundant intracytoplasmic basophilic inclusions (Fig. 3). Adrenal gland lesions were composed of clusters of necrotic adrenal cortical cells with minimal admixed acute inflammation. Gastrointestinal tract lesions were observed grossly in the stomach and small intestine (Fig. 1). The squamous epithelial portion of the stomach was multifocally hyperplastic with prominent, intracytoplasmic eosinophilic inclusion bodies (Fig. 4). One animal had a large focus of intraepithelial hemorrhage. The small intestine had extensive necrosis of gut associated lymphoid tissue (GALT) with hemorrhage and minimal epithelial necrosis. Enterocytes adjacent to the GALT had rare intracytoplasmic eosinophilic inclusion bodies. The splenic white pulp had extensive necrosis. Lung lesions consisted of patchy alveolar and interstitial edema with minimal mononuclear interstitial inflammation. Eosinophilic intracytoplasmic inclusion bodies were frequently observed in cells with the morphology of monocytes and/or type II pneumocytes. Skin lesions consisted of multiple ulcers covered by fibrin admixed with inflammation and necrotic cellular debris. The subjacent dermis was composed of organized granulation tissue and re-epithelialization was noted in some lesions from day 21.

**Figure 5:**
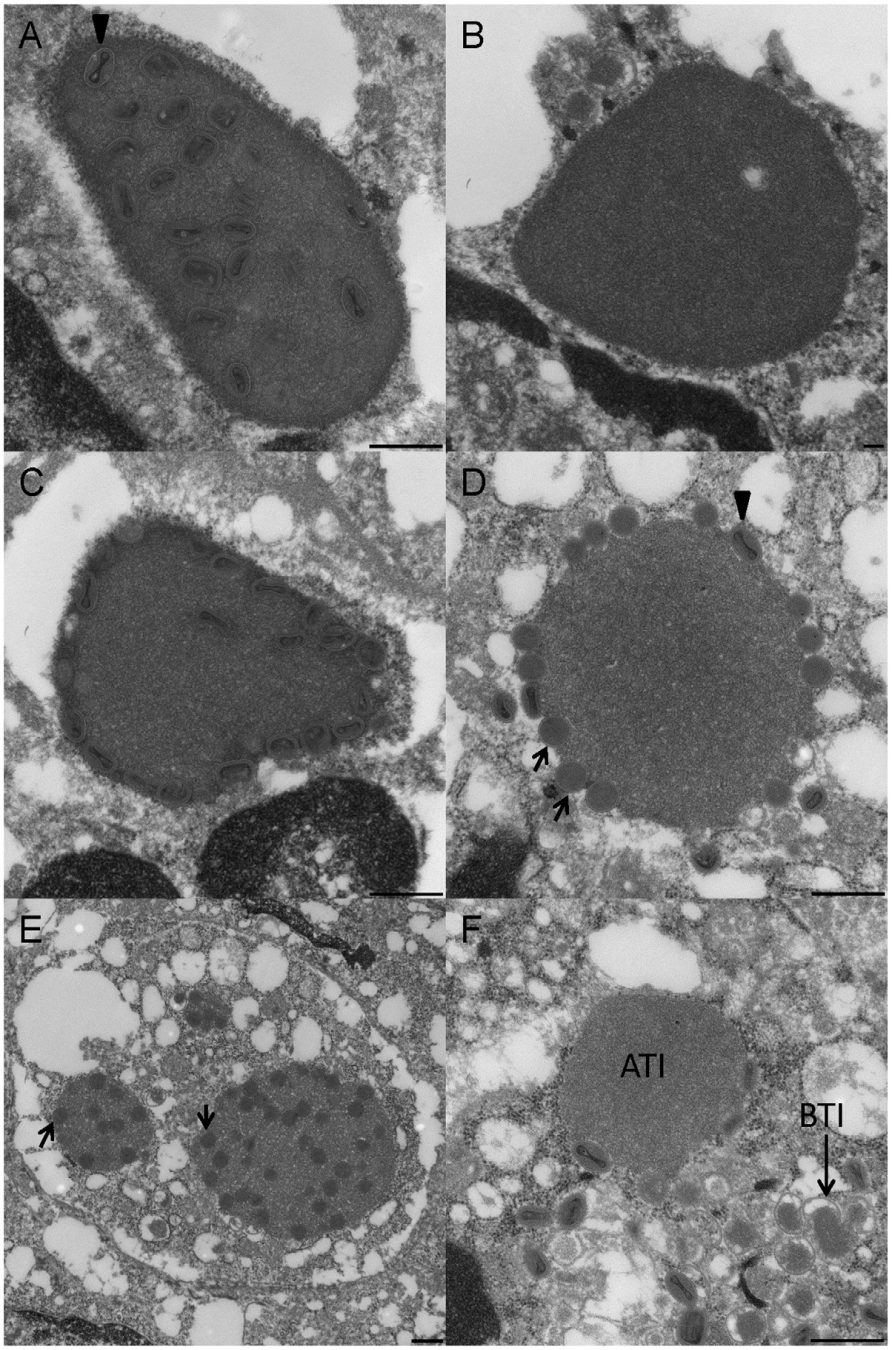
Electron microscopic examination of the inclusion bodies. Three types of ATIs were observed; inclusions containing virions throughout (Fig. 5A), inclusions without virions (Fig. 5B), and inclusions with virions at the periphery (Fig. 5C). The ATIs examined had varying morphologies that included both non-condensed and mature virions inside and/or around the periphery of the inclusions (Fig. 5D, E). B-type inclusions (BTIs) were also observed (Fig. 5F). The arrow head in figure 5A, and 5D, shows a mature volepox virion; the arrows in figure 5D, and 5E, show immature or non-condensed virions.

Electron microscopic examination of the stomach, intestine, spleen and lung revealed intracytoplasmic A-type inclusions (ATIs). Three types of ATIs were observed; inclusions containing virions throughout (Fig. 5A), inclusions without virions (Fig. 5B), and inclusions with virions at the periphery (Fig. 5C). The ATIs examined had varying morphologies that included both non-condensed and mature virions inside and/or around the periphery of the inclusions (Fig. 5D, E). B-type inclusions (BTIs or viral factories) were also observed (Fig. 5F).

### Mock Infected Animals

None of the negative control animals showed signs of disease during the study. Furthermore, all samples from these control animals were negative for volepox DNA by RT-PCR and were negative for volepox virus anti-IgG by ELISA (Table 1). The hematology values consistently remained in normal range.

## Discussion

California mice (*Peromyscus californicus*) intranasally challenged with 1.6×10^3^ PFU of VPXV in 10 µl PBS (5 µl/nostril), developed widespread lesions after a five day incubation period. The disease progression was acute, with a mortality rate of 54%; survivors began to recover by day eight and no viable virus was detectable by day 21. These data contrast with the monkeypox virus (MPXV) prairie dog model of *Orthopoxvirus* infection, where disease presentation is delayed (day 9–12) and generally persists for a longer period of time (24–28 days) before resolution. The mortality rate observed in prairie dogs infected with a similar dosage of West African MPXV is 0–25% [12], [13]. Although we did not identify a febrile period in this study, it is possible that the duration of pyrexia was very short and our sampling missed it; unpublished data show that California mice infected with a lower dose of VPXV (1.2×10^2^ PFU) did present a febrile period by day 14 pi. The weight loss and skin lesion onset may be related to anorexia due to general malaise or pain associated with oral ulceration.

The sensitivity of the NA OPXV RT-PCR is superior to viral isolation by cell culture in the detection of infection. This observation has been described previously using the E9L RT- PCR assay for detection of MPXV in prairie dog studies [12], [13], [14], [15]. All samples with CT values of 37 (minimum of 45 genomes of VPXV) or earlier had detectable cytopathic effect (CPE) in a single passage on BSC-40 cells, while samples with CT values ≥38 did not show evidence of viable VPXV. All samples from negative control animals were confirmed negative by both RT-PCR and cell culture.

The 14 kDa band was the immunodominant band observed in all survivors which manifest an immune response. This band size is consistent with the previously described envelope protein encoded by the A27 gene ortholog of vaccinia virus Copenhagen (VV-Cop). This gene has an important role in allowing mature virus to bind to cell surface glycosaminoglycans [16] and stimulates a cellular immune response [17]. Previous experiments found that mice immunized against the 14 kDa protein, and later challenged with 40 times the 50% lethal dose (LD50) of wild type VACV, did not show signs of disease and had 100% survivorship [17]. Our 36 kDa band may be the previously described 37 kDa envelope protein. This would be consistent with another study in which that protein was immunodominant in animals that succumbed to orthopoxvirus infection or had to be euthanized because of the severity of the disease [18]. The 62 kDa band is likely a major core protein encoded by the A10 gene (VV-Cop ortholog) derived from the P4a precursor. It is the most abundant core protein found in the virion and plays an important role in its assembly. It is also important in stimulating memory B-cells and the humoral immune response [17], [19]. The inflammatory monocytosis and neutrophilia in the early phase of infection was not an unexpected result given the severity of disease observed. Although mild thrombocytopenia was observed from day six through 21, it is unlikely to be the sole cause of the hemorrhage. Further analysis of platelet function and collection of clotting data throughout the duration of clinical disease would be required to accurately define the pathogenesis of the hemorrhage.

Histological changes attributable to active virus infection were seen in the internal organs of those animals that succumbed to disease. B- type basophilic intracytoplasmic inclusion bodies, also known as “viral factories”, are a typical histopathological feature of poxvirus infections, but the intracytoplasmic ATIs bodies observed ultrastructurally are not made by all members of the genus [5]. The North American orthopoxviruses, cowpox, and ectromelia have previously been shown to form ATIs [5], [9]. Our findings confirm that VPXV makes ATIs within infected cells. However, VPXV makes all three types of ATIs, which has not been reported within the genus *Orthopoxvirus*, but this could be a difference between observations made from *in vitro* versus *in vivo* systems. A-Type inclusions showed morphological variations other than the three classic types of inclusions described. In addition non-condensed and mature particles were seen inside and around the periphery of the inclusions (Fig. 5D, E). Additional studies, involving more tissue and serial thin-sections would likely provide further insight into the overall structure and composition of ATIs.

We were not able to detect an immune response in four mice. Although two of these mice (PC 024 and PC 027) presented with external and internal hemorrhagic lesions with 1×10^8^ and 6×10^7^ PFU/mL in spleen, respectively. Both mice (PC 024 and PC 027) succumbed to disease by day seven, which may be too early for IgG detection, or may be at levels below the detection limit of our assay. The other two mice (PC 010 and PC 017) which survived the infection, and were euthanized on days 42 or 49 without the production of antibodies developed single skin or tail lesions on day 7. However the CT values (38 and 40 respectively), were above the cutoff at which samples are considered positive for virus particles. Previous data indicate that lesions caused by *Orthopoxvirus* infection have very high amounts of *Orthopoxvirus* DNA which would not be consistent with the values observed from these lesion samples. Thus it is probable that these two animals were not infected. This is occasionally observed in both human vaccinations with *Vaccinia virus* where vaccinated individuals do not get a “take” [20], and in previous animal studies where *Monkeypox virus* challenged animals do not become infected and do not produce an immune response [12], [13], [15]. Further studies to evaluate both the humoral and cellular immune responses are needed in order to understand their roles in the resolution of OPXV infection.

This study is the first report describing the pathogenesis of a NA OPXV infection in a potential rodent reservoir. Previous field studies have shown that VPXV is endemic to California’s San Francisco Bay area and although lesions were observed on wild caught animals, little was known regarding the pathogenesis of this virus in mice. The California mouse is both geographically and ecologically sympatric with Pinyon mice and VPXV. The data from this study clearly indicate that *P. californicus* is susceptible to VPXV infection via the intranasal route, and that the subsequent infection can cause extreme morbidity and high mortality. It is beyond the scope of this study to characterize the pathogenesis resulting from other routes of inoculation, it is quite possible that sub-cutaneous or intra-muscular exposure could result in different disease courses. Future efforts should consider this in order to increase our understanding of *Volepox virus* pathogenesis. When comparing this model with MPXV infection in prairie dogs, we noticed that prairie dogs shed higher amounts of viable virus orally (up to 1 × 10^6^ PFU/mL), even when inoculated with lower doses of MPXV (8×10^2^ PFU) [13]. Swabs of the anus, eyes, and oral cavity had lower levels of VPXV than did the solid organ tissues, but both tissues and swabs of the infected animals had consistently high viral loads, which in all cases exceed the inoculation dose used in this investigation. Lung and liver contained the most viable virus (up to 2×10^9^ PFU/mL). This could indicate that VPXV infections may occur in wild California mice and could be transmitted between individuals in a population; however, it is noteworthy that two of the inoculated individuals that did not become infected were co-housed with two infected animals.

Several other species within the genus OPXV are rodent-borne and recognized as the causative agents of a febrile rash illness in humans. Additionally, evidence suggests that OPXV *Variola virus*, the causative agent of human smallpox, was initially a rodent-borne virus before evolving into an exclusively human pathogen [4]. The morbidity and mortality indices observed in this study are greater than previously reported in several models of OPXV disease, even those seen in highly pathogenic species (e.g., monkeypox virus and variola virus). The mouse and vole species in which VPXV is found, are non-commensal species that have relatively little contact with humans (as compared to species such as *Peromyscus maniculatus*); thus, it is possible that this virus has had little chance for transmission between the rodent hosts and humans. Due to our limited knowledge of the natural history of VPXV additional surveillance and laboratory animal studies should be pursued to address its potential risks for other animal (small mammal) and human populations.

## Materials and Methods

### Animals

Thirty one adult (17 months old) California mice were obtained from the Peromyscus Genetic Stock Center (University of South Carolina). The California mouse is sympatric with Pinyon mice, and is a congeneric relative. The mice utilized for this study laboratory raised and pathogen free,and prescreened for the absence of anti-VPXV and anti-Vaccinia virus antibodies in serum by enzyme-linked immunosorbent assay (ELISA). Additionally, blood and swabs from the oral cavity, eyes, and anus were screened by real time-PCR (RT-PCR) for the detection of VPXV DNA. Animals were pair housed in cages with aerosol filter tops. Standard mouse husbandry practices were performed during the experiment in accordance with CDC Institutional Animal Care and Use Committee (IACUC) guidelines under the approved protocol 2126-CARMOUC-A3. In addition to mouse chow all animals received oats, hay, and dried fruit as appetence monitors, as well as a plastic nest and enrichment nesting materials. Daily observations of the animal’s food consumption, activity level, and general appearance were recorded. Temperatures and weights were recorded three times a week. Hard tissues and swabs samples were taken during necropsy. All animals that lost 25% of their body weight or a total of 10 points using the following clinical scale were humanely euthanized: 2 points for decreased activity, 3 points for lethargy or innapetance, and 5 points for breathing difficulties or recumbence. Euthanasia was performed under anesthesia with 5% isoflurane gas by intracardiac exsanguination followed by cervical dislocation. On day 21, one survivor (*Peromyscus californicus* (PC) 015) was euthanized to determine if the animal had cleared infection. The rest of the California mice (10 animals) that survived infection were included in an oral rabies recombinant vaccine study, and were euthanized on days 35 (n = 2), 42 (n = 3), 49 (n = 3) or 56 (n = 2) post VPXV infection.

### Virus and Inoculum Preparation

The virus strain VPXV_USA_1985_CA was used for inoculation of animals in this study. It has been fully sequenced (Gene banks accession number: FJ807737–45), twice passaged in BSC-40 (ATCC® african green monkey kidney) cells, and purified by two sucrose cushions [21], [22]. The purified viral inoculum was diluted in phosphate-buffered saline (PBS) and titrated to verify concentration. Twenty-four *P. californicus* were inoculated intranasally (IN) with 1.6 × 10^3^ PFU of VPXV in 10 µl of PBS (5 µl per nostril) under anesthesia with 5% isoflurane gas. We choose the IN route to mimic the possibility of natural infection via contact with infected animal fluids or aerosols during social interactions. The target dose was 5×10^3^ PFU in 10 ul based on previously reported *Orthopoxvirus* animal models [12], [13], [15] but our final titeration showed that the actual inoculum was 1.6×10^3^ PFU in 10 ul. Additionally, seven animals were mock infected with 10 µl of PBS under the same conditions. The inoculation day was considered day zero. All days were recorded as days post inoculation (pi).

### Specimen Collection and Preparation

Specimens taken during necropsy (brain, gonad, kidney, liver, lung, spleen, submandibular lymph node, and adrenal gland) were collected according to IACUC and CDC standard policies. In some animals, additional organs were taken if gross lesions were observed (e.g., bladder, intestine, pancreas, skin, and/or tongue). Oral, ocular, anal, and lesion swabs were processed using the Swab Extraction Tube System (Roche), and tissue samples were homogenized using the GenoGrinder 2000 (SPEX Sample Prep) as previously described [12], [14]. DNA was extracted using Qiagen tissue kits on the BioRobot® EZ1 workstation, according to the manufacturer’s instructions.

### Viral DNA Analysis

All samples were tested in duplicate using the NA OPXV RT-PCR assay, which targets the myristylated protein gene [23]. If only one of the duplicates had a positive amplification, the sample was tested a second time. In addition to the sample, every reaction plate contained both a positive and negative control; the positive control consisted of serial 10-fold dilutions of VPXV DNA (1 nanogram –10 femtogram) and the negative control consisted of deionized, demineralized water. A sample with CT value (the cycle when fluorescence crossed the threshold) of ≤37 was considered positive.

### Virus-tissue Infectivity

BSC-40 cell monolayers were inoculated with 10-fold dilutions of sonicated tissue homogenate or swab eluate. Infected cells were incubated at 35.5°C in a 6% CO_2_ atmosphere in semi-solid medium (Roswell Park Memorial Institute medium+1% carboxymethylcellulose, 2% fetal bovine serum, and 1% penicillin/streptomycin). Cell infection was monitored microscopically by observation of OPXV characteristic cytopathic effect. At 48 hrs post inoculation, cells were stained with 2X crystal violet and plaques were counted to determine the viral titer in plaque forming units/mL (PFU/mL).

### Serological Analysis

ELISA was used for detection of NA OPXV immunoglobulin type G (IgG). We modified a previously described assay [14], using crude VPXV at 0.05 µg/well for coating the microtiter plates. Animal sera were tested at a 1∶100 dilution, 100 µl/well of a 1∶1000 dilution of anti-*Peromyscus* IgG (H+L HRP-labeled antibodies, Kirkegaard & Perry Laboratories #14-33-06) were used as conjugate. Positive and negative rabbit anti-vaccinia sera were used as assay controls on every plate. The average of all optical densities values from the BSC-40 cell lysate half of each plate, plus two standard deviations, was used to generate a cut-off value (COV). A sample’s value was considered negative if the average of the duplicates was below the COV.

Western blots (WB) were performed following the standard protocol for polyacrylamide gradient gels and polyvinylidene difluoride membranes (Bio-Rad Laboratories, CA). Fifteen µg of pure VPXV protein per well were loaded. After the protein was transferred, the membrane was placed in the BenchPro™ 4100 Western Processing System (Invitrogen, CA) for 16 hrs. Steps included: blocking for 2 hrs (nonfat dry milk, Bio-Rad #170-6404XTU), washing 3×10 minutes each (PBS+0.1% Tween 20), primary antibody incubation (1∶1000 mice serum dilution in blocking buffer) for 6 hrs, washing 3×10 minutes each, secondary antibody exposure using anti-Peromyscus IgG (H+L) AP-labeled antibodies (Kirkegaard & Perry Laboratories #15-33-06) at 1∶3000 dilution in blocking buffer for 6 hrs, and washing 3 × for 20 minutes each. Blots were developed by adding Immun-Star™ AP substrate (Bio-Rad #170-5018) for 5 minutes, followed by autoradiography exposure.

### Hematology

EDTA blood was collected from infected and non-infected animals. Utilizing the VetScan® HM5 (Abaxis, Sunnyvale, CA) we determined the values for total red blood cell counts, white blood cell counts, white blood cell differentials, platelet counts, total hemoglobin, hematocrit values, mean cell volume, mean corpuscular volume, and mean corpuscular hemoglobin concentration. The normal range of hematology values from *P.leucopus* reported previously [24] were used, as these values were similar to baseline values obtained from the pre-screening of all animals used in this study.

### Histopathology, Immunohistochemical, and Ultrastructural Analysis

Tissue specimens were fixed in 10% neutral buffered formalin, embedded in paraffin, and sectioned at 4 µm. Routine hematoxylin-eosin (H&E) stains were performed for histopathological evaluation. Immunohistochemical tests using a multi-step immunoalkaline phosphatase technique were performed on sections using a previously described technique for viruses [25]. The primary antibody used for this test was a rabbit polyclonal anti-monkeypox virus antibody known to cross react with other OPXV, including VPXV, in formalin fixed, paraffin-embedded tissue(unpublished data). Positive and negative controls were run in parallel. For ultrastructural analysis, H&E stained sections were processed for thin-section electron microscopy. Briefly, sections were prepared on-slide and processed through a graded ethanol series to rehydrate the tissue for osmium tetroxide fixation. Tissue was then block stained with uranyl acetate and rinsed with water. The sample was microwave processed with ethanol to dehydrate, followed by acetone to prepare the tissue for resin infiltration. Following four exchanges of resin, the tissue was polymerized in a final exchange of resin at 60°C. Thin sections were cut and stained with uranyl acetate and lead citrate before viewing sections on the electron microscope (Tecnai Spirit, FEI, Hillsboro, OR).

### Statistical Analysis

Wilcoxon rank-sum test (R Development Core Team. Vienna, Austria, 2008, ISBN 3-900051-07-0, URL http://www.R-project.org.) [26], [27] was used to compare weight loss, temperature, survivorship by sex, and viral titers between individuals. Day zero values were used as the baseline. A p-value less than 0.05 was considered statistically significant.
